# Supplementary material for: Exceptional origin activation revealed by comparative analysis in two laboratory yeast strains
Source: PLoS One. 2022 Feb 14;17(2):e0263569. doi: 10.1371/journal.pone.0263569 (PMC8843211; doi:10.1371/journal.pone.0263569)
Supplement: S2 File — (PDF) [file pone.0263569.s002.pdf]

Supporting Information

Supporting Tables

Table S1. Peak identification with varying minimal inter-(ssDNA) peak distance in two biological replicates for *RAD53* and *rad53* cells with A364a or W303 background.

		A364a				W303			
		RAD53		*rad53*		*RAD53*		*rad53*	
	Minimal inter-peak distance (kb)	Total # of peaks	Median inter-peak distance (kb)	Total # of peaks	Median inter-peak distance (kb)	Total # of peaks	Median inter-peak distance (kb)	Total # of peaks	Median inter-peak distance (kb)
Exp1	0.50	322	16.25	456	21.50	207	31.25	489	20.50
	0.75	317	16.75	454	21.63	206	31.38	487	20.50
	1.25	314	17.13	445	21.75	204	31.50	485	20.75
	1.75	309	17.75	441	22.00	202	31.63	483	20.75
	2.25	300	21.25	434	22.88	194	33.38	480	20.88
	2.75	271	27.00	427	23.25	186	34.88	475	21.00
Exp2	0.50	257	15.50	504	19.00	263	22.50	473	20.50
	0.75	256	15.63	501	19.25	258	23.88	471	20.75
	1.25	250	20.50	497	19.50	253	25.75	471	20.75
	1.75	246	21.00	494	19.50	249	26.50	471	20.75
	2.25	232	24.25	488	20.00	248	26.75	469	20.75
	2.75	219	28.25	480	20.50	246	27.25	466	21.00
**Ave***	**1.75**	**278**	**19.38**	**468**	**20.75**	**226**	**29.06**	**477**	**20.75**

* The average (Ave) values for the two experiments using the 1.75 kb minimal inter-peak distance parameter were shown.

Table S2. Number of origins with the indicated number of associated ssDNA peaks using a 1.75 kb minimal inter-peak distance and origins defined at varying sizes as indicated, tested for all 626 confirmed and likely origins.

Background		A364a						W303					
Strain		*RAD53*			*rad53*			*RAD53*			*rad53*		
Experiment #	# of ssDNA peaks → / Origin size (kb) ↓	0	1	2	0	1	2	0	1	2	0	1	2
1	1	582	44	0	389	237	0	541	85	0	325	301	0
	2	516	109	1	323	302	1	489	133	4	250	376	0
	3	441	151	34	269	341	16	462	149	15	211	409	6
	4	422	137	67	262	332	32	458	147	21	205	409	12
	5	416	137	73	255	333	38	456	147	23	201	406	19
	6	412	135	79	247	336	43	454	148	24	191	413	22
2	1	608	18	0	357	269	0	504	122	0	326	300	0
	2	568	58	0	275	351	0	465	161	0	258	368	0
	3	498	101	27	238	377	11	450	174	2	220	402	4
	4	465	103	58	223	383	20	444	178	4	211	408	7
	5	456	94	76	210	385	31	439	180	7	206	406	14
	6	450	94	82	202	390	34	433	185	8	197	414	15

Table S3. Primer sequences used in this study.

Primers for 2D gel DNA probes	
ARS305-L	5'-ATTCGCCTTTTGACAGGACG-3'
ARS305-R	5'-ATAACGGAGACTGGCGAACC-3'
ARS813-L	5'-GGGCAATTTACCACCTACGG-3'
ARS813-R	5'-ACGAAACTATTGGGGCCTCT-3'
ARS1006-L	5'- TCGGTTAATGAACACGTGGA-3'
ARS1006-R	5'-ATCCAACCAATGCCAACTGT-3'
ARS207.1-L	5'-GGCGGTAGCTCATTTTCTGA-3'
ARS207.1-R	5'-TTGTCCTGAAGGTGCTACCC-3'
ARS(13:269)-L	5'-CTTCGTTAAGGGCAAGACCA-3'
ARS(13:269)-R	5'-AGTTCTCCGATTGGCAGATG-3'
ARS(16:560)-L	5'-CCGTCATGCCCCAAATACTG-3'
ARS(16:560)-R	5'-AGCCAACCATTCATCCCTCA-3'
Primers for RT-qPCR	
ARS(13:269)-P1-F	5'-GATGTGATGGCCCTTTCAAT-3'
ARS(13:269)-P1-R	5'-TTTTTGTCACGTTTTGTTGAGTT-3'
ARS(13:269)-P2-F	5'-CCAATGGGCACCTGTAAGAA-3'
ARS(13:269)-P2-R	5'-TCAAAATTGCCATGCTTTTG-3'
ARS(13:269)-P3-F	5'-CTCGTGGTTTTTCGCTCAAT-3'
ARS(13:269)-P3-R	5'-AGTTCTCCGATTGGCAGATG-3'
ARS(13:269)-P4-F	5'-GAAGGGTGGCTTACCAATCA-3'
ARS(13:269)-P4-R	5'-ACATCGCACCTGAAGTGTTG-3'
ARS(16:560)-P1-F	5'-TCAGAAGCACTCCGACCTTT-3'
ARS(16:560)-P1-R	5'-AGAAGCATTTCCACAAGACGA-3'
ARS(16:560)-P2-F	5'-AATTGGGGAAGTGACACACC-3'
ARS(16:560)-P2-R	5'-GGTCATCCATTTTCAGTGTGG-3'
ARS(16:560)-P3-F	5'-ATCCCGAACATGGCATTAAA-3'
ARS(16:560)-P3-R	5'-GCACACCACCTGTCATCAAC-3'

ARS(16:560)-P4-F	5’-GCGCATGAAATCCAAGGTAT-3’
ARS(16:560)-P4-R	5’-CCAGCATCTTCGAGACAACA-3’
ARS(16:560)-P5-F	5’-GATAGGGCGTTGATCTTGGA-3’
ARS(16:560)-P5-R	5’-GCGTCGAAATCTCTTGGAAC-3’
ARS(16:560)-P6-F	5’-GCGCATGAAATCCAAGGTAT-3’
ARS(16:560)-P6-R	5’-CCAGCATCTTCGAGACAACA-3’
Primers for ChIP-qPCR	
ARS305-F	5’-GCCTTTTGACAGGACGATAA-3’
ARS305-R	5’-CTCCAAAGAAGGCTCTGAAA-3’
ARS306-dist-F	5’-TGTTTTATCCGGTCATTCCA-3’
ARS306-dist-R	5’-CGCCAGAAGCAAACATTTC-3’
ARS306-F	5’-TCGTGAGGAAGGAAAGTGTT-3’
ARS306-R	5’-TGTTTTTCGACAAAAGTTGC-3’
ARS313-F	5’-TGAGGGCAGTTTAAGTGCTA-3’
ARS313-R	5’-GTAGGCGCTTTTATCTCCAG-3’
ARS501-F	5’-GCAGCTCCAAAAGAAAGGAA-3’
ARS501-R	5’-GGCGTTAGGTAATGCTCAAT-3’
ARS603-F	5’-TTGTTAAGTGCTGGGAGAAC-3’
ARS603-R	5’-CTGGGAAAGAGAATTCAACC-3’

Supporting Figures Captions

Figure S1. Fork distance averaged from two experiments based on the aggregated ssDNA plots. Two-way ANOVA test followed by Tukey's multiple testing for all pair-wise comparisons was performed. Error bars denote standard deviation.

Figure S2. Cell cycle analysis. (**A**) Budding indices (percentage of budded cells) of cells synchronously entering S phase in the presence of 200 mM HU after release from the G1 block. (**B**) Flow cytometric analysis of the number of cells (Y-axis, arbitrary unit) with varying amounts of DNA content (X-axis, arbitrary unit) when undergoing the same treatment as above. "Asyn" stands for asynchronous cells prior to G1 block by α-factor. The "1C" and "2C" DNA content, corresponding to G1 and G2 phase cells, respectively, are as indicated. A dashed line is drawn across the samples at the "1C" position to indicate the subtle but detectable shift of the peak to the right on the X-axis.

Figure S3. Gene-expression profile of control samples- DUN1 and GAPDH. Relative gene expression in S phase cells exposed to 200 mM HU compared to G1 control (S/G1) for DUN1, whose expression is induced by HU and dependent on Rad53. GAPDH served as a negative control.

Supporting File Caption

Data File S1. Complete list of all activated origins with their firing status in the *RAD53* vs. *rad53* cells in the A364a and W303 background. Each origin is also marked by the origin category defined in the text.

Figure S1

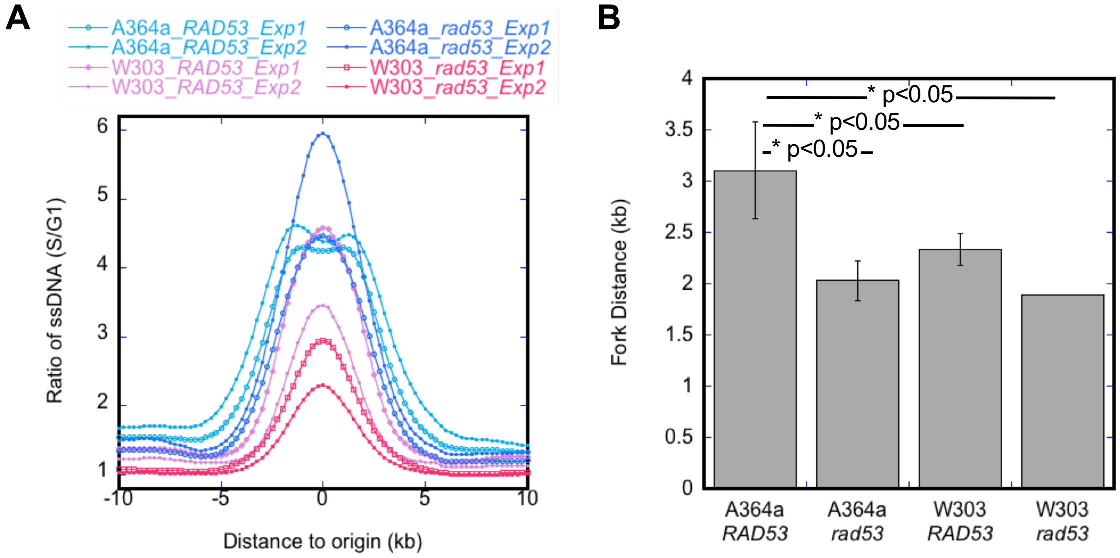
A
A364a_RAD53_Exp1
A364a_RAD53_Exp2
W303_RAD53_Exp1
W303_RAD53_Exp2
A364a_rad53_Exp1
A364a_rad53_Exp2
W303_rad53_Exp1
W303_rad53_Exp2
Ratio of ssDNA (S/G1)
Distance to origin (kb)
B
* p<0.05
* p<0.05
* p<0.05
Fork Distance (kb)
A364a RAD53
A364a rad53
W303 RAD53
W303 rad53

Figure S2

A

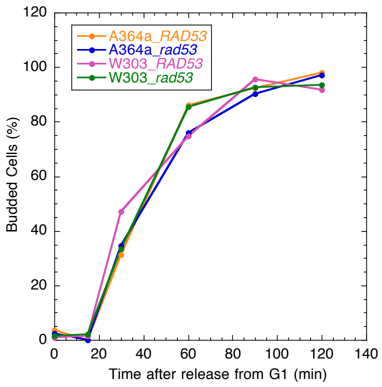

A364a_RAD53
A364a_rad53
W303_RAD53
W303_rad53
Budded Cells (%)
Time after release from G1 (min)
0
20
40
60
80
100
120
140

B

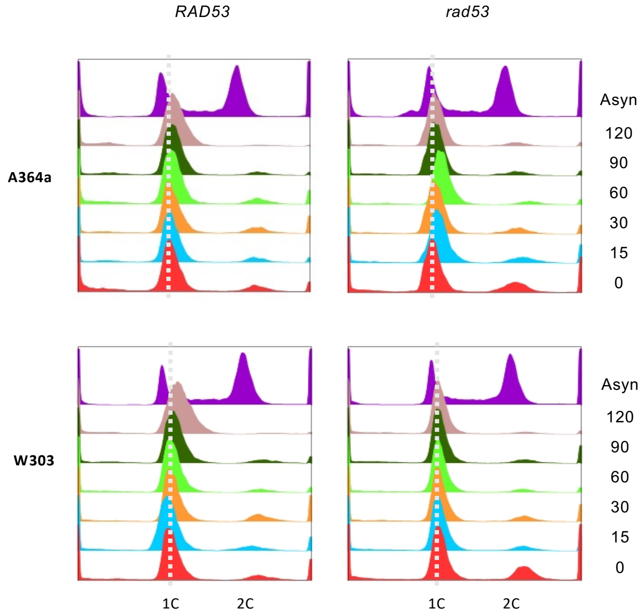

RAD53
rad53
A364a
W303
Asyn
120
90
60
30
15
0
1C
2C

Figure S3

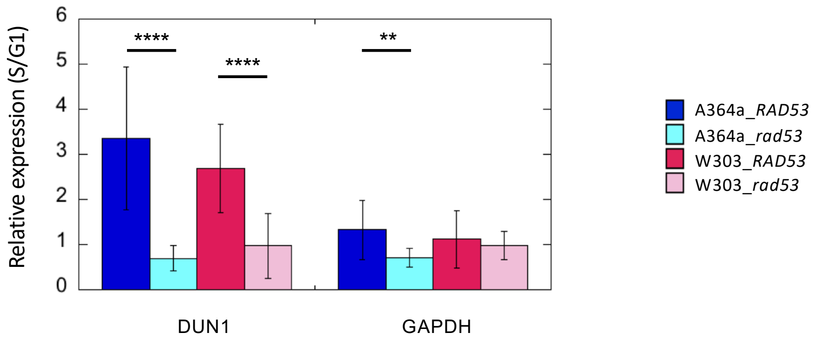

Relative expression (S/G1)
6
5
4
3
2
1
0
****
****
**
DUN1
GAPDH
A364a_RAD53
A364a_rad53
W303_RAD53
W303_rad53
