# Supplementary material for: Exceptional origin activation revealed by comparative analysis in two laboratory yeast strains
Source: PLoS One. 2022 Feb 14;17(2):e0263569. doi: 10.1371/journal.pone.0263569 (PMC8843211; doi:10.1371/journal.pone.0263569)
Supplement: S1 Raw images — (PDF) [file pone.0263569.s003.pdf]

Anti-Rad53 Western Blot

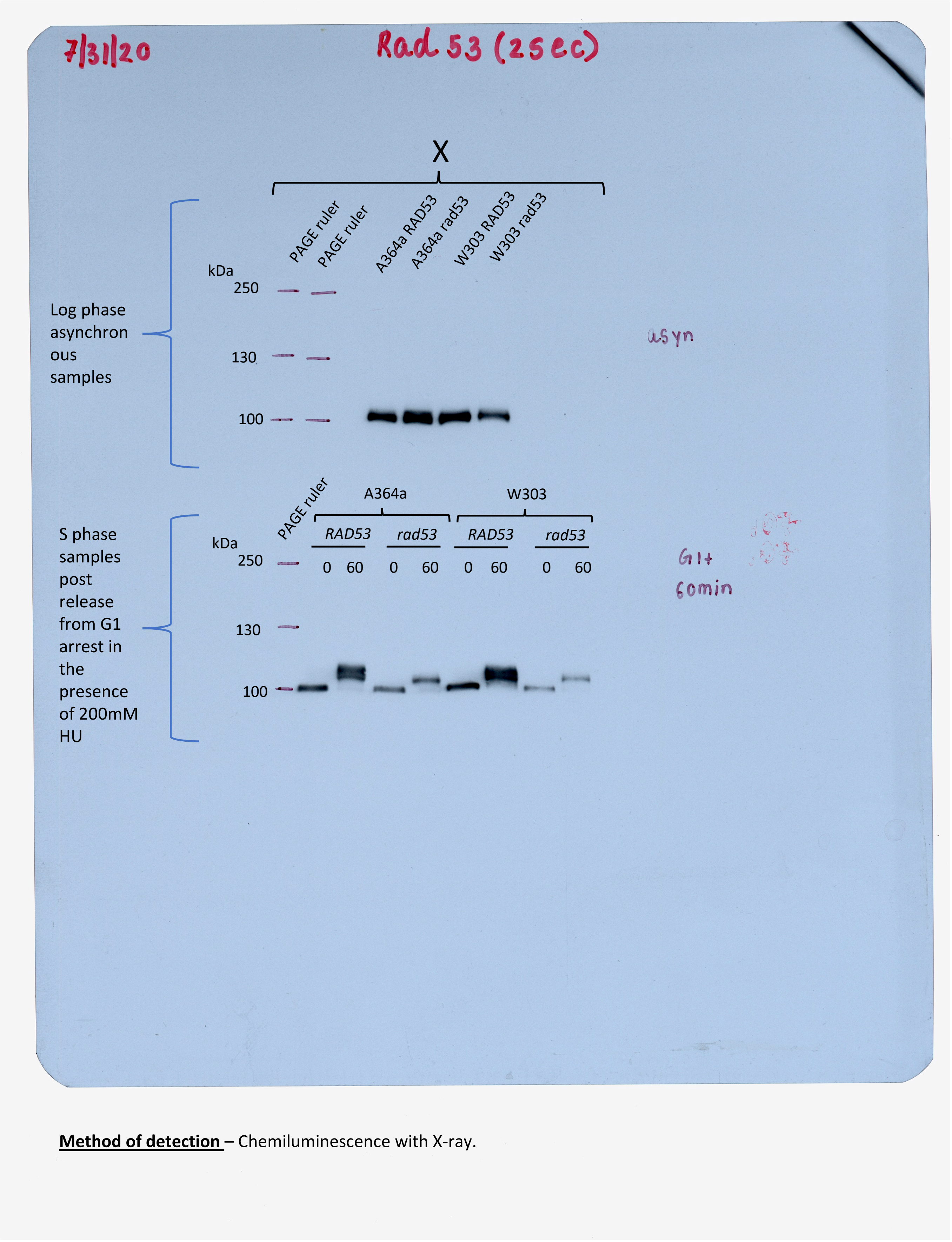

Method of detection – Chemiluminescence with X-ray.

Ponceau Stained Rad53 Blot

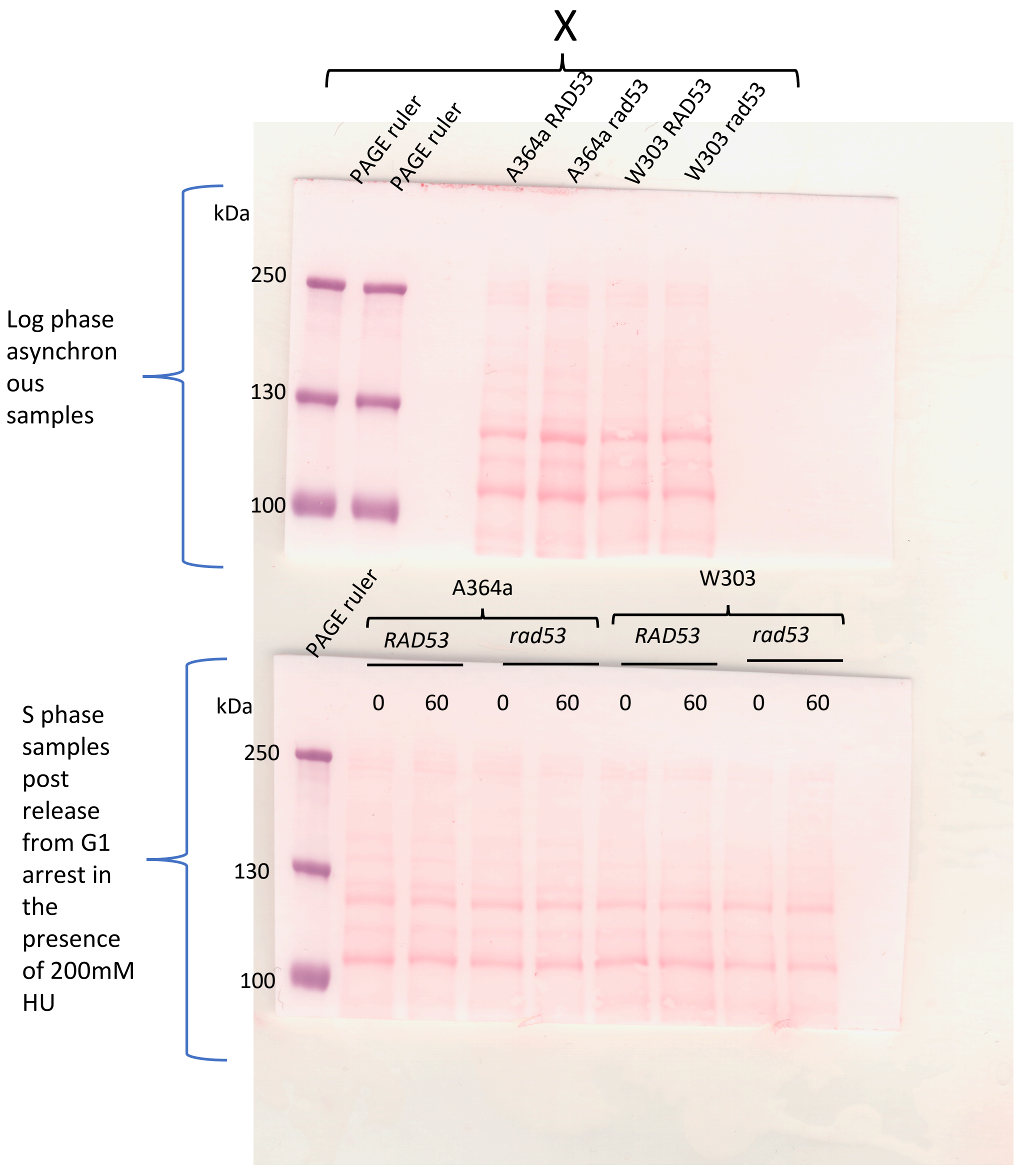

<u>Method of detection</u> – Scanning the ponceau stained blots.

Anti-Vma1 Western Blot

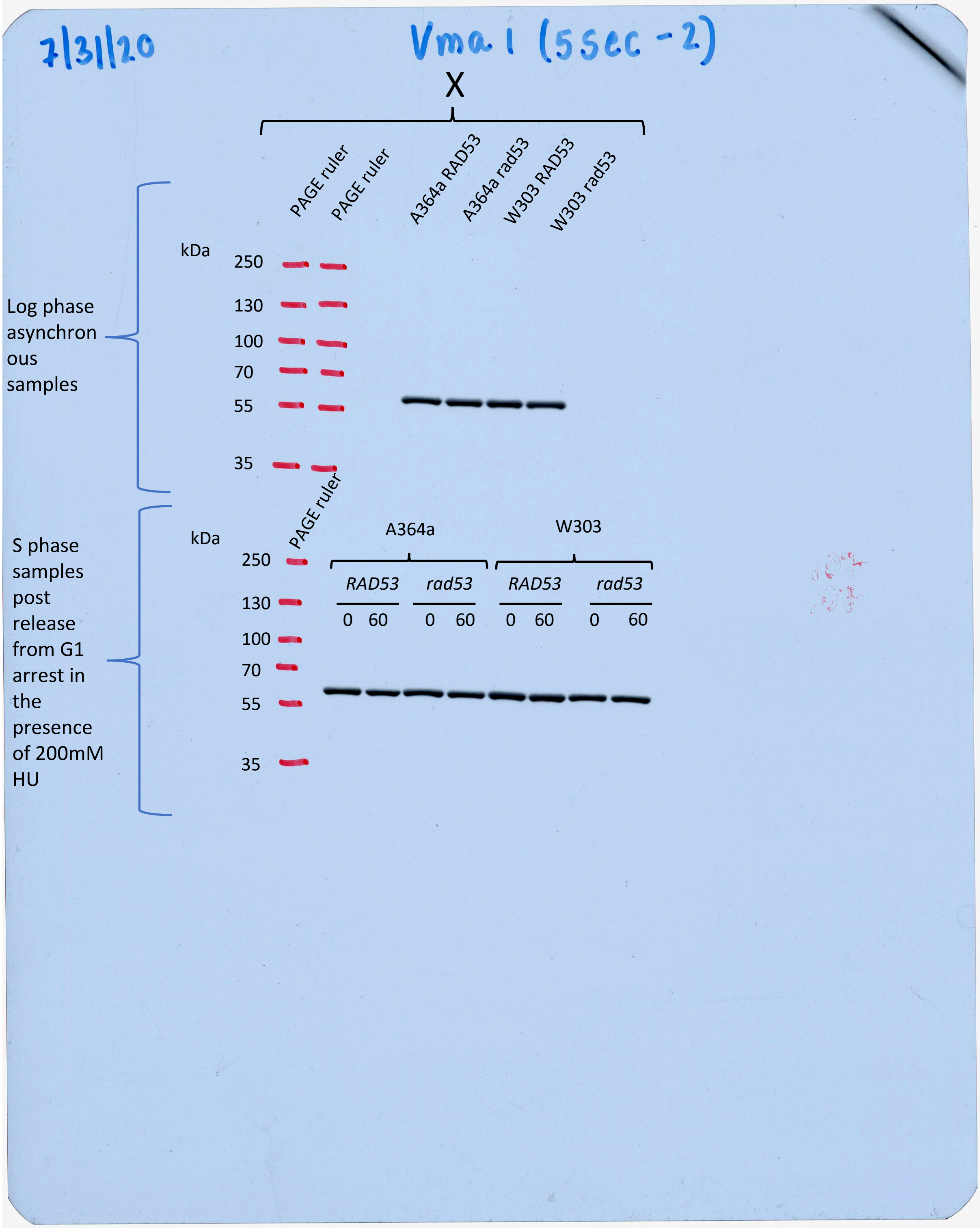

Method of detection – Chemiluminescence with X-ray.

Ponceau Stained Vma1 Blot

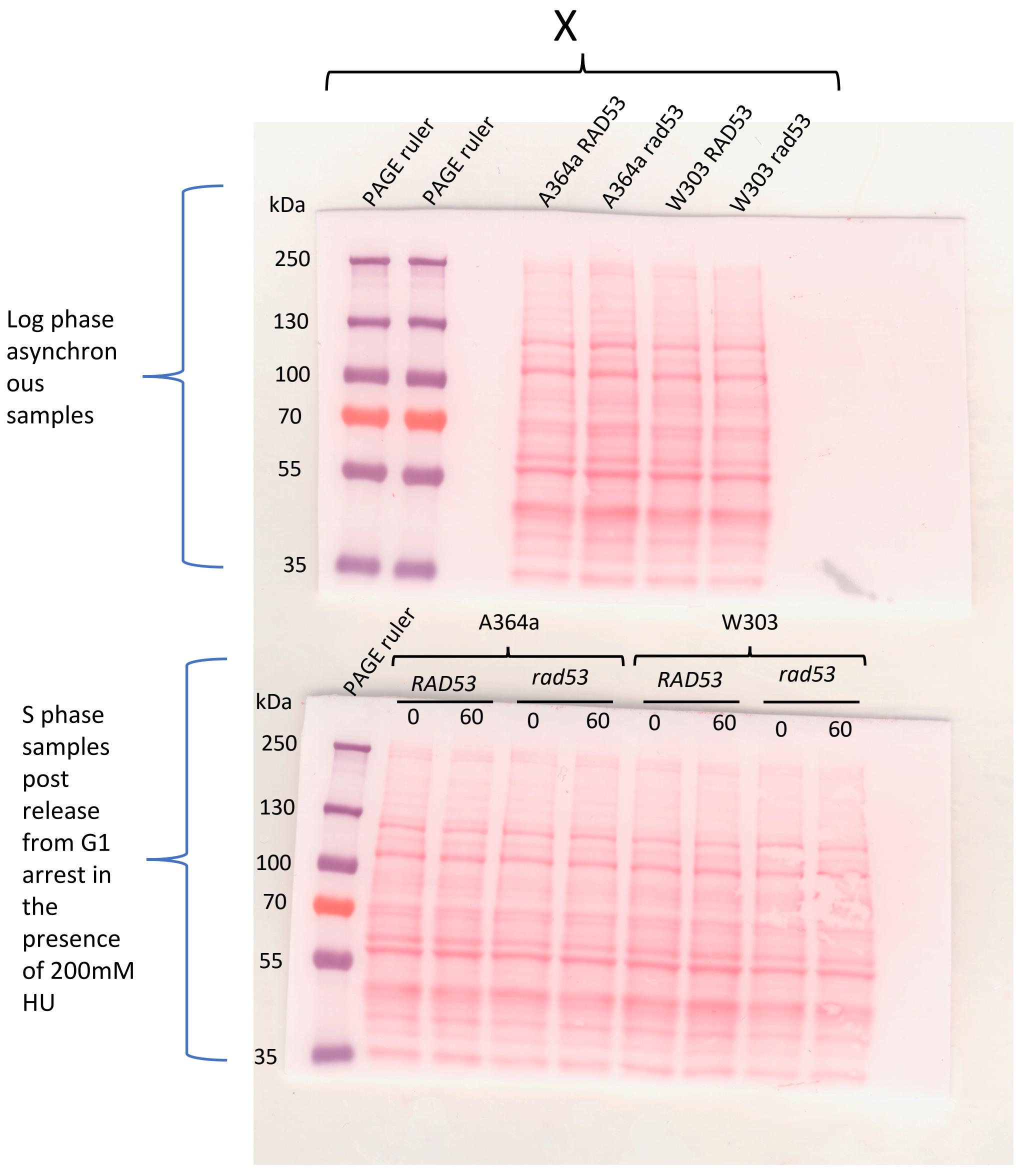

Method of detection – Scanning the ponceau stained blots.

ARS305 2D Gel scanned Image

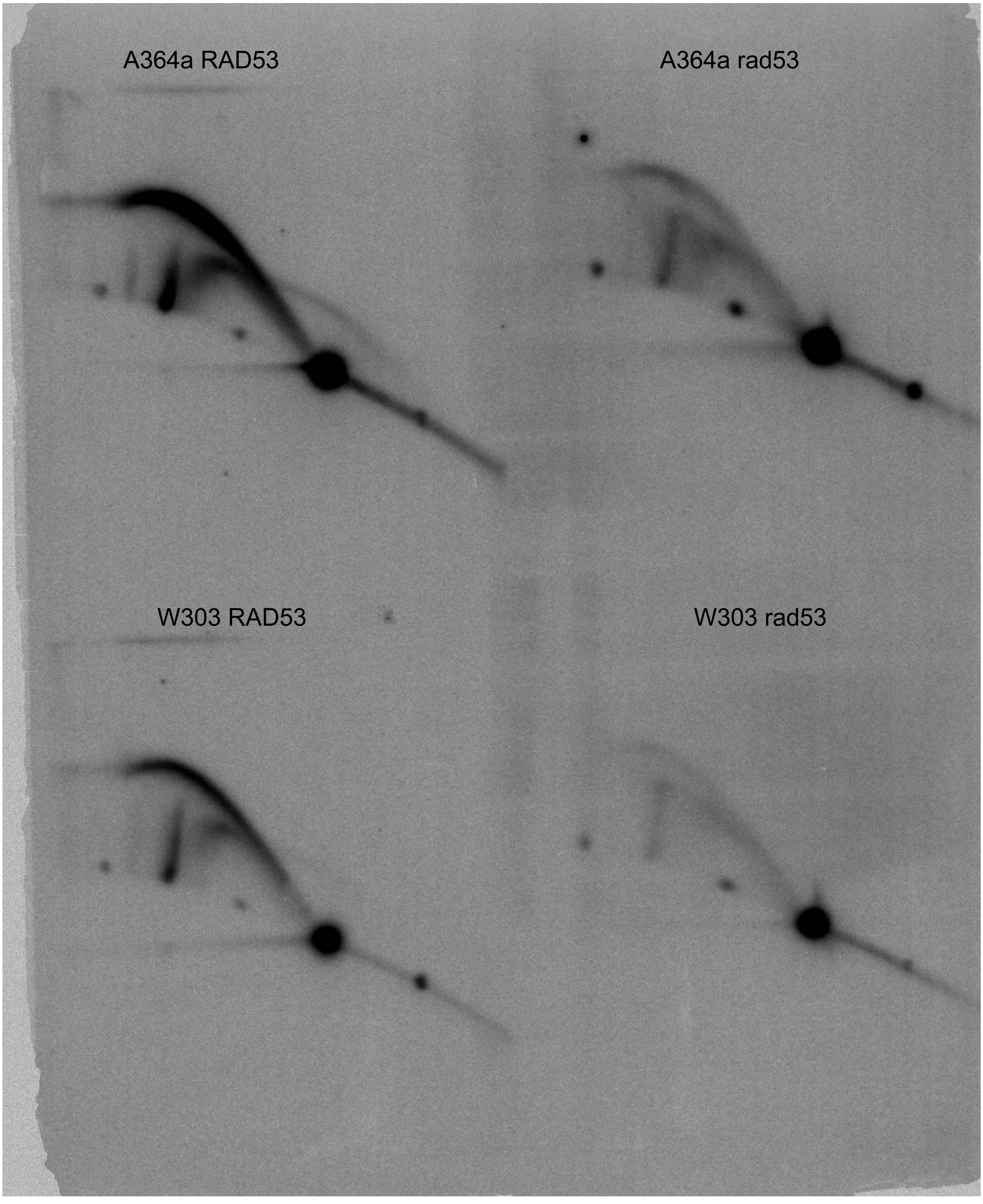

Method of detection –Phospho imaging of P32 labelled blots.

ARS813 2D Gel

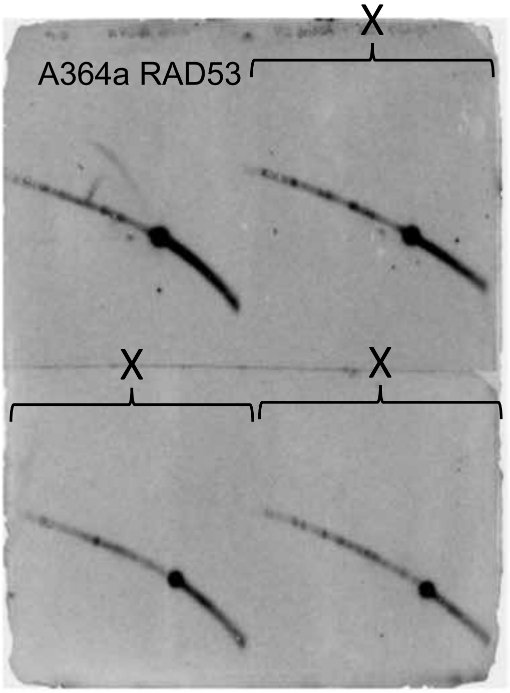

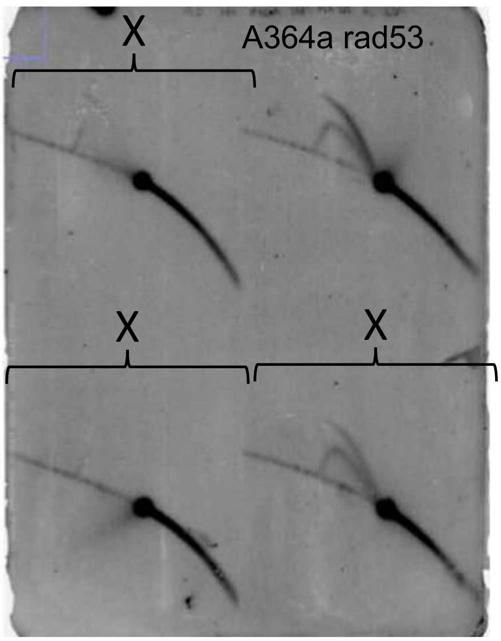

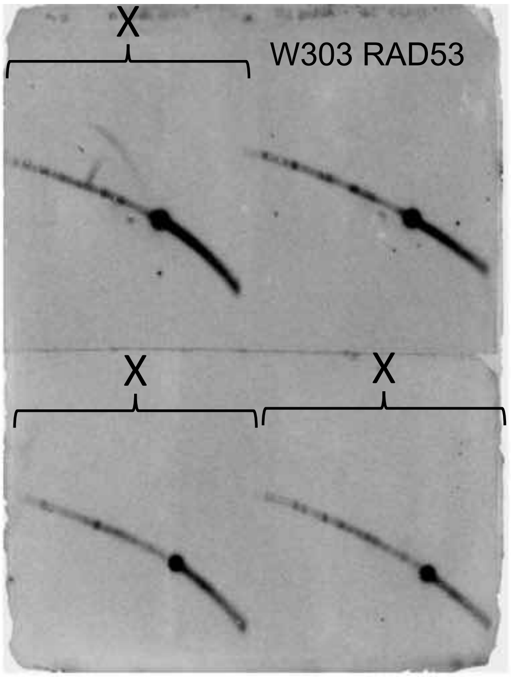

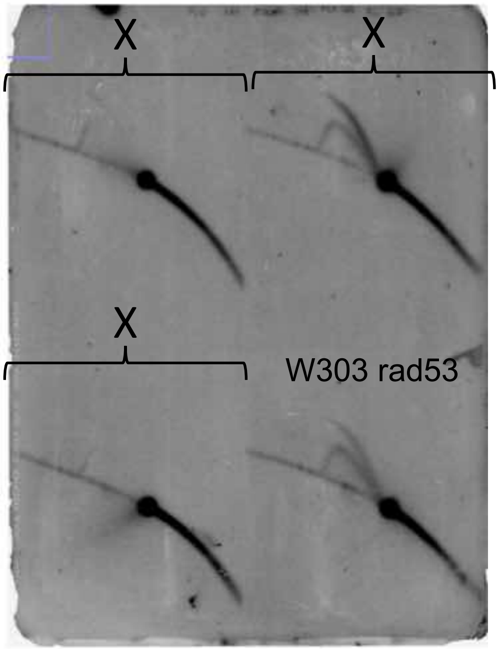

Method of detection –Phospho imaging of P32 labelled blots.

ARS207.1 2D Gel

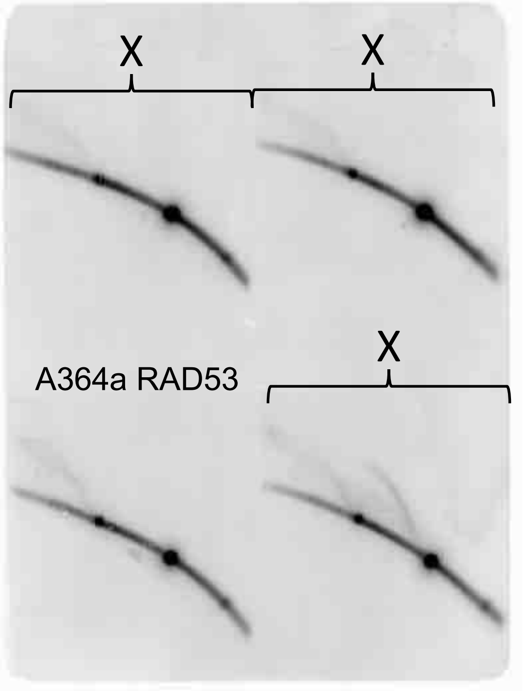

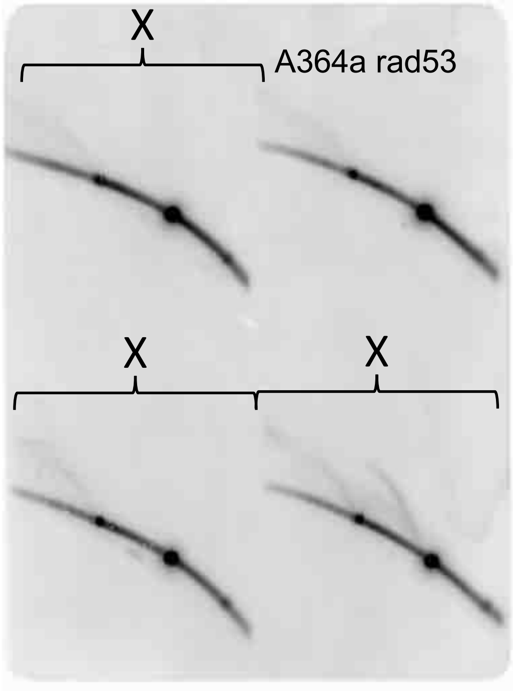

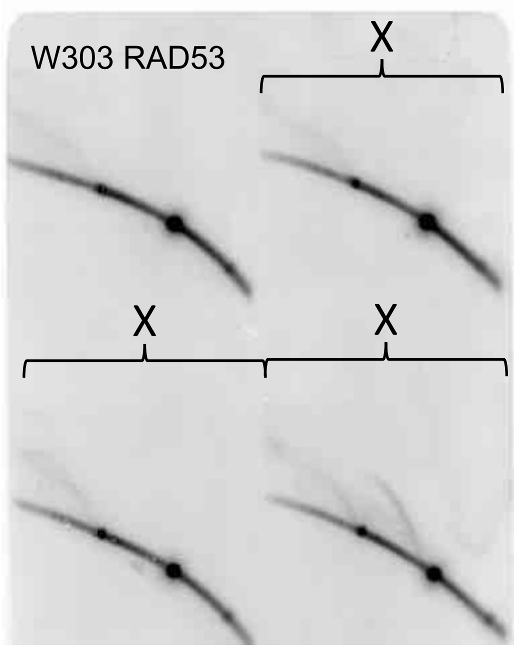

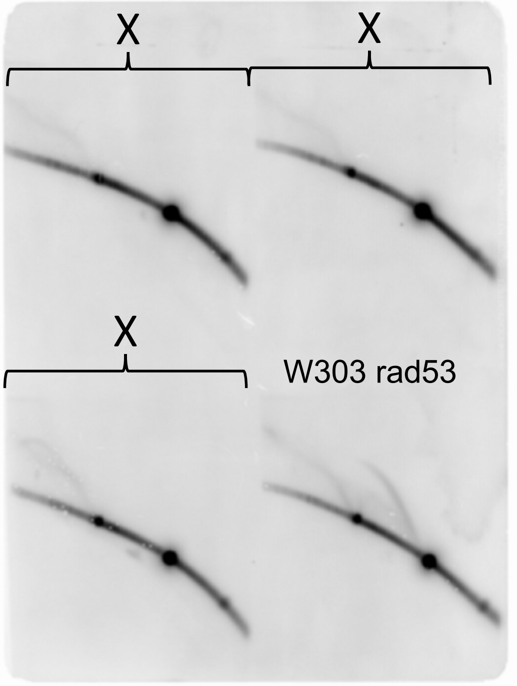

<u>Method of detection</u> –Phospho imaging of P32 labelled blots.

ARS1006 2D Gel

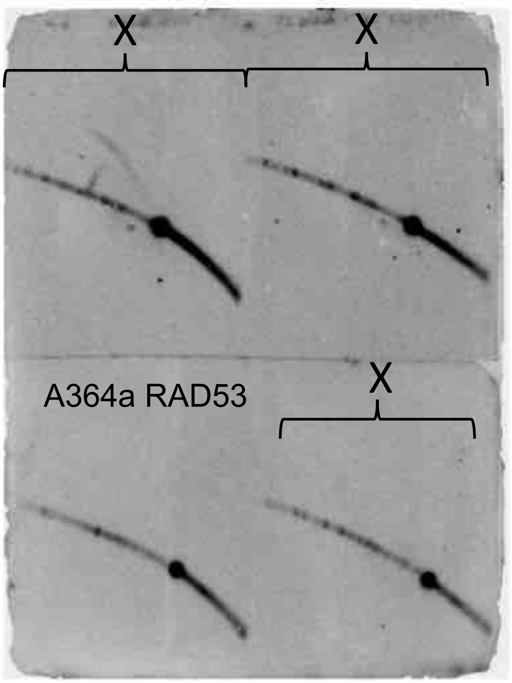

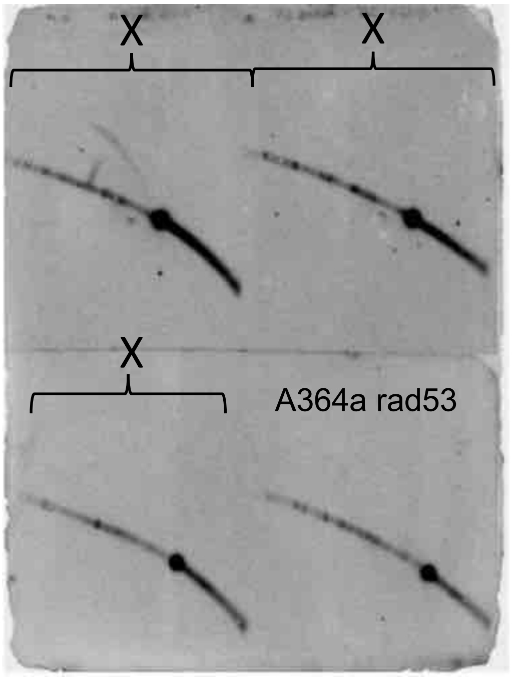

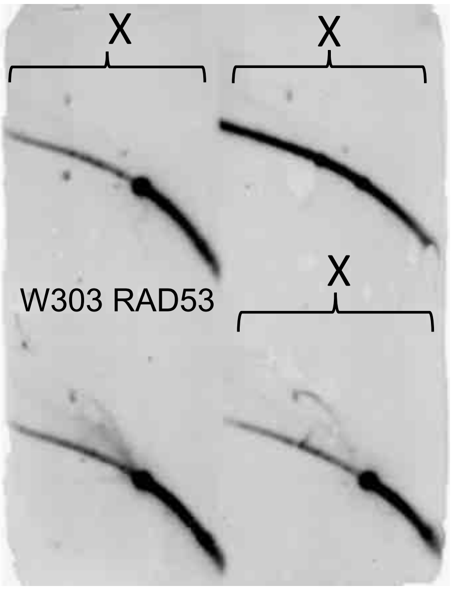

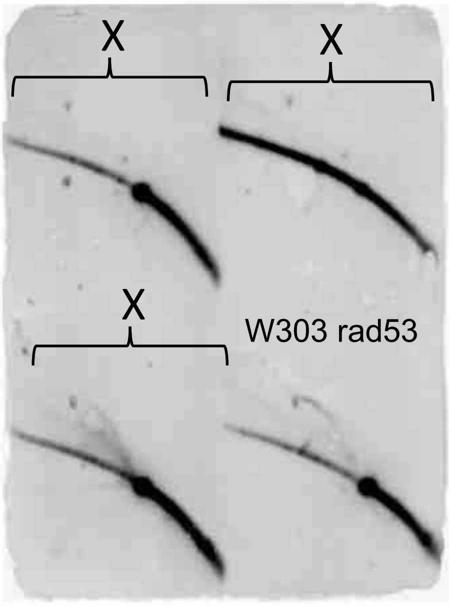

Method of detection –Phospho imaging of P32 labelled blots.

ARS13.269 2D Gel

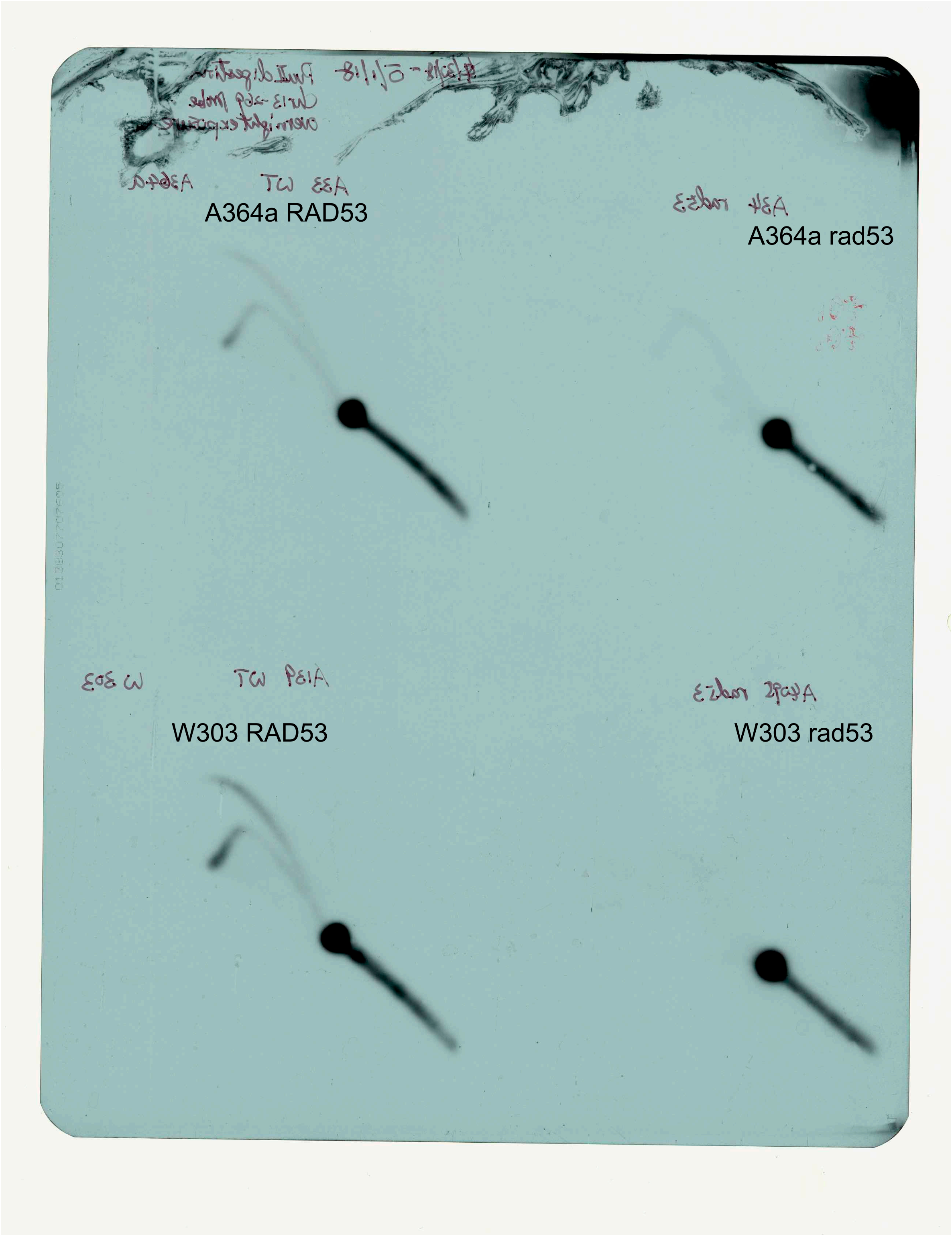

<u>Method of detection</u> – Hybridization of P32 labelled membrane with X-ray film followed by detection using radiography.

ARS16.560 2D Gel

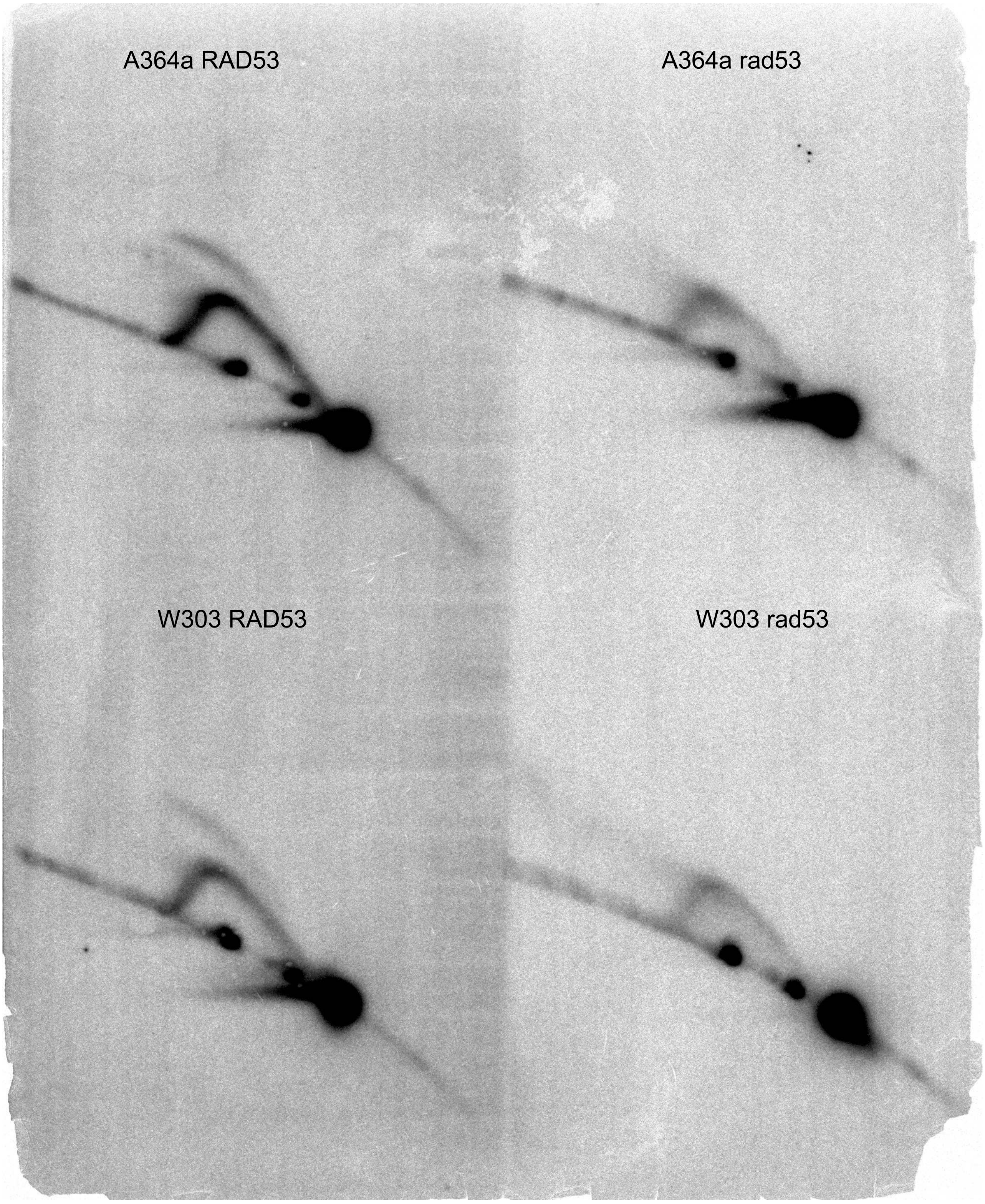

Method of detection –Phospho imaging of P32 labelled blots.
