# Supplementary material for: Exceptional origin activation revealed by comparative analysis in two laboratory yeast strains
Source: PLoS One. 2022 Feb 14;17(2):e0263569. doi: 10.1371/journal.pone.0263569 (PMC8843211; doi:10.1371/journal.pone.0263569)
Supplement: Attachment — Submitted filename: rebuttal.pdf [file pone.0263569.s004.pdf]

Response to review by the AE

All reviewers considered this study to be a significant addition to the field. However, they also point out the need for further experimental corroboration of hypotheses, further consideration of the validity of hypotheses, and additional elaboration of the findings reported in the paper. We thank all reviewers for their encouragement and positive feedback on how to improve our work.

The major issues that must be remedied are noted below:

1. The experiments need to be extended to answer some additional basic issues including the association of Rad53 to the origins. This issue should be addressed using ChIP-qPCR of initiation factors at several well-defined origins on chromosome III in the RAD53 and rad53 strains of both W303 and A364a. Done. Please see new figure 3 and the text (line# 211-229).
2. The genomic studies that suggest the presence of Rad53-dependent origins should be by performing RT-qPCR on RNA extracted from HU- treated RAD53 and rad53 cells. Done. Please see new figure 6D and the text (line# 345-357).
3. The investigators should describe the experimental reasons for the choice of the two strains used and compare the known characteristics of these strains. Done. Please see text (line# 111-126).
4. The authors performed replication origin identification at the genomic level. This information must be provided to the reader in supplementary information (either directly or through a link) Supplementary file S1 summarizes all origin information.
5. The authors should also use to DeOri database. Indeed, ARSseq was curated at DeOri. We have now referenced the database.
6. The authors need to point out that autophosphorylation is inferred rather than directly shown or provide alternative data demonstrating autophosphorylation. We refrained from suggesting that the phosphorylation we observed was due to autophosphorylation by Rad53.
7. The Remus studies refer to an in vitro analysis. Is it appropriate to apply these data to an in vivo situation? Furthermore, the authors need to modify their hypothesis for a negative regulation of fork movement given the possibility of alternative interpretations. We think so. Much of the molecular events during replication were first characterized in vitro, as exemplified by the ordered assembly of the pre-RC.
8. Provide a rationale for the use of the Vma1 protein for Western blot normalization. We added this information in the figure legend for figure 2.
9. The authors need generally to discuss their results and speculations to a greater degree in the text as discussed by the Reviewers. We have expanded both the result and discussion sections as directed (line# 394-423).
10. In Discussion, the authors need to differentiate between the dNTP effect on fork stalling vs. fork collapse. See previous point.
11. Studies were not conducted in the absence of HU to my understanding. Please eliminate indications to the contrary or provide additional data. Corrected.

12. Authors should compare the analysis from the Bielinsky lab who compared the genome-wide replication profiles of rad53-1 mutant are described in as much as the differing strain backgrounds permit. The study by Raveendranathan, Bielinsky, et al. utilized a microarray containing 424 previously identified origins (not truly genome-wide) to compare wild type vs. checkpoint mutants for origin activation. It by and large recapitulated the finding that checkpoint mutation allows late origins to prematurely activate in HU. And indeed, as the reviewer pointed out, due to differences in strain background and methodology we found a direct comparison to be tenuous.
13. Please state the reference sequence in Figure 4. Done. We stated in the Methods section, and now also in Figure 4 (the new Figure 5) legend, that the S288C reference genome R64.2.1 was used.
14. Please edit the text carefully to eliminate grammatical errors. Done.
15. Please address all other issues raised by the Reviewers in a point-by-point response. See below.

Response to reviewers

Reviewer #1: In this manuscript, the authors used a published single-stranded DNA mapping protocol to identify active replication origins in two different laboratory strains of S. cerevisiae (W303 and A364a) after their release from G1 into hydroxyurea in the presence or absence of an active Rad53 S-phase checkpoint factor. The majority of active origins were shared between the two strains and these origins also shared the same overall response to the status of the Rad53-dependent S phase checkpoint. However, the level of ssDNA at origins and the distance traveled by the fork were significantly more reduced in the W303 rad53 mutant strain and correlated with a more robust phosphorylation of Rad53 in the W303 RAD53 strain. A number (71) of origins were only active in W303 or A364a. Comparison of published genome DNA sequencing data between the two strains found that sequence polymorphisms in the consensus ACS element were unlikely to account for the differences. The authors believed that origin activation likely occurred because of the presence of multiple mismatched ACS elements in the DNA sequences. Finally, the authors identified 6 origins that they characterized as Rad53-dependent, in that they were only active in RAD53 but not in rad53 strains. Interestingly, 5 of these origins also overlapped with an ORF, in contrast to the intergenic location of most origins. The lack of origin activation in rad53 cells was not a consequence of transcriptional interference, as published data indicated that the genes in which the origins were located were transcribed at a lower level in rad53 cells compared to RAD53 cells.

Overall, this is an interesting and well documented study, with many of the conclusions from genomic data validated by 2D agarose gel analysis. One of the most remarkable findings was the observation that despite the similar number of origins activated in rad53 between the two strains, W303 was significantly more sensitive to the reduced checkpoint than A364a, showing reduced levels of ssDNA at all origins and reduced spreading of ssDNA from origins. This is shown most dramatically in Figure 1C. The authors speculated that this might be due to the lower level of Rad53 in the W303 rad53 mutant, which in turn led to lower levels of Rad53-P. The striking differences between the two strains need to be much more fully discussed in the text. What could account for the lower activation of all origins in the W303 rad53 mutant? Is this a consequence of the role of Rad53 in the initiation of DNA replication that is independent of checkpoint

regulation, and if so, by what mechanism? Is the loading of initiation factors altered at origins, and is Rad53 binding to origins affected? Is there an effect on nucleotide pools in this particular rad53 mutant? Some of these questions could be addressed using ChIP-qPCR of initiation factors at several of the well defined origins on chromosome III in the RAD53 and rad53 strains of both W303 and A364a. This would help to address questions on the differential effects of the rad53 mutation in the two strains, and perhaps more generally uncover some new insights into the relationship of the levels of Rad53 to replication initiation.

We thank the reviewer for these suggestions. Please see our response to point #1 in AE's comments.

Other comments:
1. The identification of RAD53-dependent origins in 5 ORFs represents a very interesting finding. The authors should confirm the published genomic data on these genes by performing RT-qPCR on RNA extracted from HU treated RAD53 and rad53 cells. Some speculation on the possible function of RAD53 at these genes would also be beneficial. Please see response to point #2 in AE's comments.
2. The text needs to be carefully edited as there were some missing sentence parts. Done.

Reviewer #2: Identification and characterization of replication origins are essential for a better understanding of the molecular mechanism of DNA replication. The authors conducted experimental procedures to perform the differential dynamics of origin activation in the A364a and W303 Saccharomyces cerevisiae strains. The authors also found the groups of “Rad53-unchecked” and “Rad53-checked” origins by the cooperation of origin usage in wild type and the rad53 mutant cells. The identification of replication origins from the genome-wide and the analysis of a new class of origins would provide new insights into the replication mechanism of S. cerevisiae.

Major comments:

1.Authors should describe the detailed reasons for choosing the strains used in the study. What’s the phylogenetic distance of A364a and W303 S. cerevisiae strains? The authors mentioned that “Genetic variation in diverse laboratory strains can manifest in distinct physiological properties”. What’s the phenotypic difference between these two strains? What’s the significant phenotypic difference associated with DNA replication between these two strains? Done. Please see response to point #3 in AE's comments.

2.The authors identified the “Rad53-dependent origins”, however, the author needs to clarify that these “Rad53-dependent origins” are not strain-specific. We state in the text that some of the Rad53-dependent origins are in fact strain-specific, though the two origins we characterized by 2D gel and gene expression are not.

Minor comments:

1.The authors used the ARS records of the OriDB database. OriDB is one of the well-known yeast replication origin databases. I recommended the authors also use the DeOri (DOI: 10.1093/bioinformatics/bts151), a database for eukaryotic replication origins, to verify and

support your experimental results. Please see response to point #5 in AE's comments.

2.The authors performed the identification of replication origins of two yeast strains by genome-wide level. I'd like to suggest the authors attach the detailed information of identified replication origins, including chromosomal position and replication origin sequences, to the supplementary file, which will help the authors and other researchers to further explore the mechanism of DNA replication. Please see response to point #4 in AE's comments.

3.I'd like to recommend some of recent papers related to DNA replication origins in Saccharomyces cerevisiae genome for your kind reference.

Wang, D, Lai FL, Gao, F. Ori-Finder 3: a web server for genome-wide prediction of replication origins in Saccharomycescerevisiae. Briefings in Bioinformatics 2020, doi: 10.1093/bib/bbaa182
Wang D, Gao F. Comprehensive Analysis of Replication Origins in Saccharomyces cerevisiae Genomes. Frontiers in Microbiology, 2019, 10: 2122.
Peng C, Luo H, Zhang X, et al. Recent advances in the genome-wide study of DNA replication origins in yeast. Frontiers in Microbiology, 2015, 6(FEB): 117.

We thank the reviewer for bringing to our attention these publications. We will certainly use them for our future studies.

Reviewer #3: Comments on the manuscript PONE-D-21-08460 (Peng et al. entitled 'Exceptional origin activation revealed by comparative analysis in two laboratory yeast strains').

In this manuscript, authors performed a comparative analysis of replication origin activation in two yeast strains W303 and A364a when cells were exposed with hydroxyurea. They also analyzed the effect on origin activation of the checkpoint kinase, Rad53, by combining the checkpoint-deficient Rad53-K227A mutant. They found that the activation of replication origins are similar in both strains. However, they find strain-specific origin usage. Although some of strain-specific origins have SNPs, they suggested that the SNP is not the reason of the strain-specificity. They also suggested that the difference of origin usage is partly depends on the activity of Rad53. Finally, they identified a new class of origins that are active only when Rad53 is functional.
Although the study is descriptive rather than analytical, some of their findings are interesting and are worth for the publication in the PLoS ONE. However, I feel there are some points that should be clarified before acceptance.

Major points
1. Authors insist that the Rad53 levels are different between W303 and A364a (p. 10). It does not seem that there is a big difference between wildtypes. I also wonder why authors use Vma1 as a control rather than ponceau staining. Moreover, it is known that Rad53 autophosphorylates when it is activated. If authors want to indicate the difference of Rad53 activity in this situation, kinase assay would be the best way. The way simply compare band intensities might be inappropriate, because some of them are not autophosphorylated, it is difficult to extract only autophosphorylated ones, and the extent of autophosphorylation must be considered if you want to say the activity. Please see response to points #6 and #8 in AE's comments. We agree with the reviewer that the difference between Rad53 levels in WT strains is moderate. Yet they were reproducibly seen. We further corroborate with the ChIP-PCR data of Rad53 at origins.

In discussion, they suggested that Rad53 has the function on fork progression by referring the recent Remus Lab's results (ref #35). However, this is an in vitro results obtained by adding the aphidicolin to inhibit DNA polymerase function. I wonder this situation can be applied directly to cells treated with HU. Moreover, in W303 rad53-K227A cells, which has lower levels of Rad53 protein than corresponding A364a cells, fork did not travel longer than wild type nor A364a rad53-K227A (Fig. 2D). Therefore, their interpretation (p10 middle part) and discussion (p17 middle part) seem incorrect for me. Please see response to points #7 and #9. We now provide a more in-depth discussion on the replication fork phenotype in the mutant cells, which we believe clarified the questions the reviewer had.

2. Authors say, 'This is consistent with the notion that checkpoint failure causes replication forks to collapse shortly after initiation from the origin.' (p9, bottom). I think replication forks stall rather than collapse by dNTP shortage in HU-treated cells even in checkpoint-deficient cells. If there are evidences that support authors description, they should be clearly shown here. Shorter fork travel could be caused by more origin firing. Simply because less dNTPs are available when more origins fire. We agree with the reviewer that dNTP pool level should be considered here and we added that in our discussion. We have previously demonstrated that replication forks collapse in the checkpoint-deficient cells after HU treatment using density transfer as a readout (Feng et al. 2009). We now added this reference for clarification (line # 194).

3. Authors say, 'We conducted a comparative analysis of origin activation, with and without DNA replications tress by HU, using genomic ssDNA mapping in two common laboratory yeast strains.' (the first sentence of Discussion). I do not think this is the case. Authors did not do anything without HU. We thank the reviewer for pointing out this mistake. We corrected the statement (line # 360-361).

4. Authors do not refer the previous analysis form Bielinsky lab (Raveendranathan et al. (2006) EMBO J. 25:3627-39), in which genome-wide replication profiles in rad53-1 mutant are described. If authors can compare both results, it would be great. I admit this might be difficult, because the strain background is different (Bielinsky lab uses the strain BF-15D). Please see response to point #12 in AE's comments.

5. Authors concluded that SNPs cannot explain the strain-specific origin activation. Authors can obtain solid conclusion by introducing such SNPs into other strains directly to see the effect of SNPs. We agree with the reviewer and will implement these experiments in our future studies.

Minor points

In Figure 4, authors show the difference in the DNA sequence in the strains. What is the reference sequence? S288c in the Saccharomyces Genome Database, or not? We stated in the Methods section, and now also in Figure 4 (the new Figure 5) legend, that the S288C reference genome R64.2.1 was used.

Some sentences are broken. For example,
Page 5, lines 18-19.
Page 16, line 3. 'replications tress' should be 'replication stress'

There could be more. We have gone through the entire document after revision and eliminated all grammatical errors.
