# Supplementary material for: Exceptional origin activation revealed by comparative analysis in two laboratory yeast strains
Source: PLoS One. 2022 Feb 14;17(2):e0263569. doi: 10.1371/journal.pone.0263569 (PMC8843211; doi:10.1371/journal.pone.0263569)
Supplement: Attachment — Submitted filename: responsetoreviewers_20211224.pdf [file pone.0263569.s005.pdf]

We thank all three reviewers for their time and insightful comments. Below we address the additional comments from reviewers 2 and 3 regarding the first revision (in blue text).

Reviewer #1: (No Response)
Reviewer #2: Almost all the comments have been addressed properly. However, I still have some minor comments as follows.

1. DeOri database was mentioned in the main text of the manuscript, but the relevant reference was not cited.
We apologize for the oversight. The reference has been added.

2. Some words in Figure 6D were obscured, such as ARS(16:560), 4.5 on the Y axis.
Thank you for the pointing this out. We have remade the graphs after performing new analyses.

3. The words A364a and W303 shall be of uniform size in Figure 1B.
We thank the reviewer for pointing this out. We have fixed it in the revision.

Reviewer #3: Comments on the manuscript PONE-D-21-08460R1 (Joshi et al. entitled 'Exceptional origin activation revealed by comparative analysis in two laboratory yeast strains').

In this revised version, authors addressed most of issues raise by me. I also appreciate authors' effort to improve their work. However, I feel some issues are not fully addressed in a satisfactory manner. Moreover, I noticed that newly added data include other issues that cannot be published as it is. Specific points are follows.

5. (just a comment) 'Revised Manuscript with Track Changes' does not include proper 'track-change' info, which made review process more laborious.

Thank you for the comment and sorry for the inconvenience. In this new version we have highlighted the modified text in blue (those original text to be removed are additionally marked by ~~strikethrough~~ marks) to distinguish from the remainder of the text and facilitate the review process.

1. (Previous Major point #1) Authors responded as 'We agree with the reviewer that the difference between Rad53 levels in WT strains is moderate. Yet they were reproducibly seen.' If it is reproducible, authors should show the average of Rad53/Vma1 value obtained from several repeats and statistical (p) value. Authors also need to explain why Vma1 is appropriate as an internal control.
We thank the reviewer for the suggestion. Instead of showing data from one representative experiment we are now including a plot with averaged Rad53/Vma1 ratios and statistics from multiple measurements. Indeed, the results indicate that the there is only minor difference between the Rad53 levels in the two strain background. Therefore, we have now modified the conclusion from these experiments. The text now reads as follows.

"The results demonstrated that phospho-Rad53 (Rad53-P) was readily detected in WT cells in both A364a and W303 (Fig. 2D). The checkpoint mutation reduced the levels of both Rad53K227A and Rad53K227A-P to a similar degree in both strain background (Fig. 2D). These observations suggested that the checkpoint operated at similar levels in A364a and W303, and thus unlikely caused the global reduction of origin activation in the W303_*rad53* cells."

Accordingly, the section in Discussion that originally summarized these results were also modified as follows.

"Arguably the most striking phenotype associated with the W303 background is that W303_*rad53* cells showed global reduction of origin activation. ChIP-qPCR analysis revealed that this phenotype was not due to decreased level of initiation protein (Cdc45) binding, but rather probably due to increased Rad53 binding at origins. ~~Because the ssDNA at replication forks is induced by HU, we directed our investigation at the Rad53-mediated checkpoint response. We detected a higher level of phosphorylated Rad53 in W303 cells than in A364a. Moreover, Rad53 phosphorylation was greatly reduced by the checkpoint mutation in both W303 and A364a. Finally, though the level of the mutant Rad53 protein was lower in W303 than in A364a there was a higher level of the mutant protein at the origins in the W303 background.~~ We speculate that these results could be explained by the multiple roles of Rad53 in regulating origin activation and replication fork progression. In addition to down-regulating late origin activation, it has been shown that Rad53 is also important for coordinating leading strand synthesis with DNA unwinding [53]. Moreover, a recent study reported that Rad53 limits excessive unwinding of DNA at replication forks by the replicative helicase CMG (Cdc45-MCM-GINS) complex [54]. Therefore, the net impact of Rad53 at origins during initiation and subsequent fork movement is likely difficult to delineate. Nevertheless, our data provided a clue to how increased Rad53 binding at W303_*rad53* origins may underlie global suppression of origin activation. ~~The stronger activation of wild type Rad53 in W303 cells may cause increased inhibition of replication fork progression relative to the A364a background. On the other hand, the lower Rad53K227A protein level in W303 cells would cause more Rad53-checked origins to activate in W303 compared to A364a."~~

Regarding Vma1 as a loading control, we had in the past tried using GAPDH and ß-actin which are well known and widely used loading controls. But, the available antibodies for these gave us non-specific bands hence they were not reliable to use and perform normalization. Vma1 is a subunit of the vacuolar V-ATPase whose expression does not show variations in different strain background in our experiments. Hence, we considered Vma1 a comparable metabolic gene and an appropriate control, as GAPDH and ß-actin.

2. (Previous Major point #2) Authors seems not to distinguish 'stall' and 'collapse' rigorously. In my understanding, when dNTPs short, first, replication forks stall. If cells do not have proper checkpoint machineries at this point, integrity of replisome then will be lost and replication fork cannot be restart anymore (= collapse) even after the supply of dNTP is back, as shown in Labib lab. In addition to this, If CMG itself is collapse by accident, replication fork will be collapsed. In this manuscript, most of ss region detected by authors

in rad53 mutant are first caused by fork stall, rather than collapse of the replication fork, I guess.

We agree with the reviewer that the molecular basis of ssDNA formation in the checkpoint mutant is not being resolved by our study. We do not believe that our method is capable of distinguishing between ssDNA produced by fork stalling vs. collapse. One of the reasons is if DNA synthesis is uncoupled from unwinding by CMG, a situation that could lead to fork collapse, those ssDNA gaps due to unwinding without synthesis will not be detected in ssDNA labeling as the template strands would anneal back with each other during DNA isolation. As stated previously we have also shown that *rad53* cells are not capable of resuming synthesis even after HU removal, indicating replication fork collapse and failure to restart (Feng et al. Genetics 2009). In the current revision, we consistently described the phenotype in the *rad53* mutant as such, for instance, in the sentence below. But we agree with the reviewer that *rad53* may experience a broad range of fork defects upon, or potentially before, nucleotide depletion. To give a more rounded view of the observed reduction of fork progression in *rad53* cells compared to WT, we now modified the text as follows. In this sentence we define replication fork collapse more accurately as the failure to resume synthesis. We hope this sufficiently addresses the reviewer's concern.

"...the extent of ssDNA formation in both *rad53* mutants in the A364a and W303 background was reduced compared to their *RAD53* counterparts (Fig. 2B). This observation was consistent with *rad53* cells accumulating aberrant replication fork intermediates when encountering HU, ultimately resulting in replication fork collapse or the failure to resume synthesis [26, 47]."

3. Newly added ChIP data in Figure 3 do not agree with previous data obtained in many labs (Araki, Bell, Labib labs etc.). That is: in G1 phase, Cdc45 does associate with early origins but neither late origins nor non-origin region. In this experiment, authors do not include negative control and use conventional PCR rather than qPCR to measure the amount of ChIP-ed DNA. This might be the cause of the discrepancy. The way to show results in this figure are also not acceptable, because all statistical descriptions including that on error bars are missing. I also am not sure Rad53 can ChIP replication origins. No one have shown such association, as far as I know.

We thank the reviewer for the suggestion to transition to qPCR from ChIP-PCR. We also took to heart the consideration to use a negative control to normalize data. We now provide ChIP-qPCR data for multiple origins, using a non-origin locus as control. We have also performed statistics (One-way ANOVA followed by Tukey's multiple comparison) to indicate significant changes between samples.

With regards to the concern over the Rad53 ChIP experiment, we would like to cite two recent independent studies – Can et al, Molecular Cell 2018 and Sheu et al, bioRxiv 2021 where the authors reported Rad53 binding to origins by ChIP-seq. But the reviewer is correct possibly assuming in that the binding of Rad53 is less robust than other initiation proteins. Its detection does require more rigorous crosslinking for detection as detailed in our Methods section.

4. Figure 6D. As in Figure 3 all statistical details are missing, and I cannot understand why authors put a break in Y axis. What does it mean the value of Y axis: 'relative expression (S/G1)'? I cannot find any explanation in the manuscript. Error bar in P1 (red) from ARS(16:560) which reaches to 5, seems to be a bad joke.

The reviewer's comment is well taken. We have added statistics for both gene expression and ChIP-qPCR data. We also provided better explanation for the calculation of "relative expression (S/G1)", *i.e.*, the ratio of gene expression in S phase sample to that in G1 control sample. The break in Y axis allows better presentation of the low values (between 0 and 3 in the graph shown to the right) that would have been compressed on a scale without break.

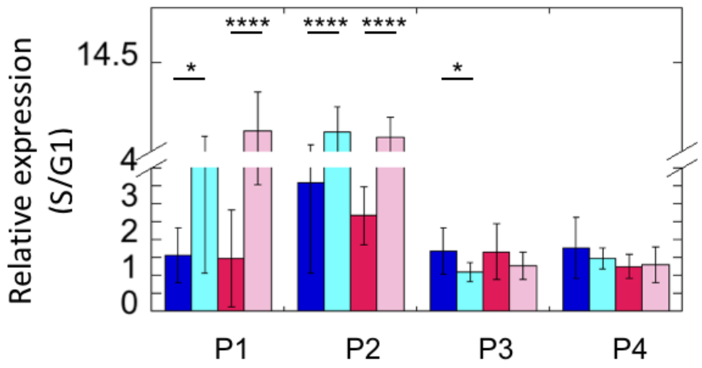

We have now performed additional replicate experiments in an effort to reduce experimental error. In addition, we had removed measurements where two of the triplicate wells (for either G1 or S phase samples) failed PCR amplification from the final calculations. Previously, the single value from the triplicate was used to calculate % Input. These practices resulted in significant reduction of error bars for the P1 locus at ARS(16:560). However, we note that significant error still persisted, such as at the P1 locus of ARS(13:269) shown above in the *rad53* mutant. However, statistical analysis demonstrated significant difference between WT and *rad53* cells.
