# Supplementary material for: Exceptional origin activation revealed by comparative analysis in two laboratory yeast strains
Source: PLoS One. 2022 Feb 14;17(2):e0263569. doi: 10.1371/journal.pone.0263569 (PMC8843211; doi:10.1371/journal.pone.0263569)

We would like to address reviewer 3's comments regarding the second revision below (in blue text).

Reviewer #3: Comments on the manuscript PONE-D-21-08460R2 (Joshi et al. entitled 'Exceptional origin activation revealed by comparative analysis in two laboratory yeast strains').

In the left panel of Figure 6D (a bar graph of ChIP results on ARS(13:269), authors break graph just above 4 and show only one scale in the upper part of the graph. As a result, it is not possible to read the values of several results and the top of the bar of P1 from A364a_rad53 are not shown. These issues must be fixed before publication.

Thank you for catching this mistake and we apologize for the oversight. We realized that the ARS(13.269) plot in Figure 6D does not require an axis break. We have replaced it with a new plot (see below). This change does not require modification of the manuscript text regarding this result. However, we did correct a few typos in the figure legends, which are highlighted in blue in the document named "MainText_20220120_trackchange.docx".

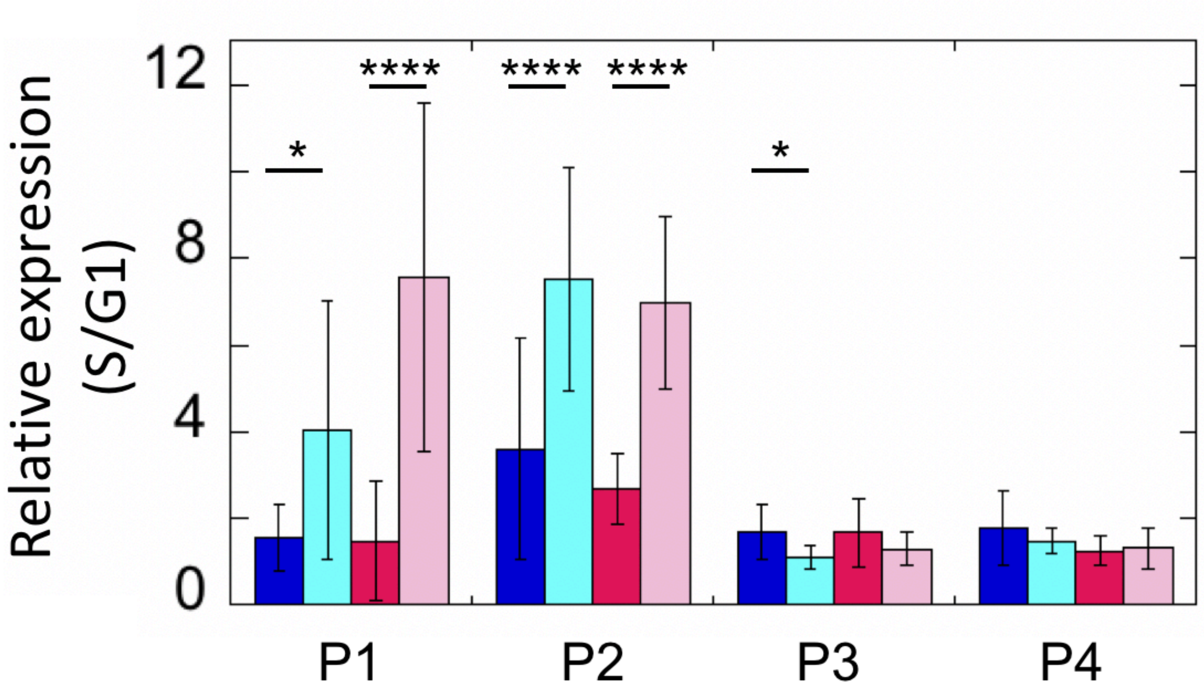

Supplement: Attachment — Submitted filename: responsetoreviewers_20220120.pdf [file pone.0263569.s006.pdf]
